# Trends in Regionalization of Care for ST-Segment Elevation Myocardial Infarction

**DOI:** 10.5811/westjem.2017.8.34592

**Published:** 2017-09-11

**Authors:** Renee Y. Hsia, Sarah Sabbagh, Nandita Sarkar, Karl Sporer, Ivan C. Rokos, John F. Brown, Ralph G. Brindis, Joanna Guo, Yu-Chu Shen

**Affiliations:** * University of California, San Francisco, Department of Emergency Medicine, San Francisco, California; † University of California, San Francisco, Philip R. Lee Institute for Health Policy Studies, San Francisco, California; ‡ National Bureau of Economic Research, Cambridge, Massachusetts; § Alameda County Emergency Medical Services Agency, Oakland, California; ¶ University of California, Los Angeles-Olive View Medical Center; Geffen School of Medicine, Los Angeles, California; || San Francisco Emergency Medical Services Agency, San Francisco, California; # University of California, San Francisco, Department of Medicine, San Francisco, California; ** Naval Postgraduate School, Graduate School of Business and Public Policy, Monterey, California

## Abstract

**Introduction:**

California has led successful regionalized efforts for several time-critical medical conditions, including ST-segment elevation myocardial infarction (STEMI), but no specific mandated protocols exist to define regionalization of care. We aimed to study the trends in regionalization of care for STEMI patients in the state of California and to examine the differences in patient demographic, hospital, and county trends.

**Methods:**

Using survey responses collected from all California emergency medical services (EMS) agencies, we developed four categories – no, partial, substantial, and complete regionalization – to capture prehospital and inter-hospital components of regionalization in each EMS agency’s jurisdiction between 2005–2014. We linked the survey responses to 2006 California non-public hospital discharge data to study the patient distribution at baseline.

**Results:**

STEMI regionalization-of-care networks steadily developed across California. Only 14% of counties were regionalized in 2006, accounting for 42% of California’s STEMI patient population, but over half of these counties, representing 86% of California’s STEMI patient population, reached complete regionalization in 2014. We did not find any dramatic differences in underlying patient characteristics based on regionalization status; however, differences in hospital characteristics were relatively substantial.

**Conclusion:**

Potential barriers to achieving regionalization included competition, hospital ownership, population density, and financial challenges. Minimal differences in patient characteristics can establish that patient differences unlikely played any role in influencing earlier or later regionalization and can provide a framework for future analyses evaluating the impact of regionalization on patient outcomes.

## INTRODUCTION

Despite the relative decrease in the incidence of ST-segment elevation myocardial infarction (STEMI) over the past decade, STEMI still comprises about 25–40% of acute myocardial infarction incidents.^1^ STEMI patients can be diagnosed early in their course with 12-lead electrocardiography (ECG) performed in the field by paramedic personnel and transmitted wirelessly to the hospital. This technology has accelerated the delivery of STEMI care, such as improving target times to treatment and emergency medical services (EMS) transport.^2^,^3^

In an effort to optimize access to and delivery of care, policies from the American Heart Association (AHA) have advocated for establishing regional systems and networks.^4^ Implementation of regionalized care has numerous potential benefits, including greater likelihood of primary percutaneous coronary intervention (PCI) treatment, improved door-to-balloon times, and lower rates of mortality, stroke, and heart failure.^2^

Several regions in the United States have begun adopting innovative successful efforts to regionalize STEMI care, demonstrating feasible outcomes by integrating inter-hospital transfers and prehospital protocols;^2^ however, barriers to system implementation have existed over the last decade.^2^,^3^ Characterizing the trends of regionalized STEMI care systems can help provide an understanding of the elements that encourage or prevent regionalization in order to better inform areas looking to restructure or improve the organization of current EMS services and health systems.

In California, EMS care is divided into 33 separate local EMS agencies (LEMSAs), and while all LEMSAs must have their EMS plans abide by general state EMS authority mandates, no specific mandated protocols exist to define regionalization of care.^5^ Recent steps have been taken to improve this, including development of protocol parameters to be incorporated into all local EMS protocols for common, severe medical complaints such as chest pain.^5^ The variations between LEMSAs and their regionalization-of-care protocols provide an opportunity to study the spectrum of how STEMI regionalization of care has been implemented.

Previous work studying the effects of STEMI regionalization has not fully addressed demographic and clinical patient and hospital characteristics,^6^–^9^ potentially missing some underlying differences. Our descriptive characterization of the population will help identify any drastic differences between counties, ruling out potential causes of patient outcome differences outside of the adoption of regionalization protocols such as racial differences in PCI access and mortality outcomes.^10^–^12^ We aimed to perform a careful examination of underlying demographic characteristics for potential differences to provide the necessary groundwork for analyzing the impact of regionalization on patient outcomes in California. Therefore, the two major objectives of this paper were to 1) describe STEMI regionalization-of-care trends in California; and 2) examine the differences in patient demographic, hospital, and county trends between earlier vs. later regionalized counties across the state.

**Population Health Research Capsule**

What do we already know about this issue?

Regionalization of care is a system that directs ambulances with STEMI patients to hospitals with PCI capabilities, which has been shown to improve treatment times.

What was the research question?

What are current trends in regionalization of care for STEMI patients in California?

What was the major finding of the study?

We found hospital and potential geographic differences between earlier and later regionalized counties.

How does this improve population health?

Our findings identify potential barriers to establishing regionalized systems of care, and may provide a framework for regionalization of care in other regions.

## METHODS

### Study Design

We collected survey responses from all California EMS agencies (details below), and linked the survey responses to 2006 California non-public hospital discharge data to understand the patient distribution at baseline. We obtained hospital information from annual surveys conducted by the AHA and annual reports submitted through the Healthcare Cost Report Information System maintained by the Centers for Medicare and Medicaid Services. We used county-level data from the U.S. Census for population demographics. We calculated the numbers of STEMI patients each year using *International Classification of Diseases, Ninth Revision* codes 410.0 through 410.6 and 410.8^13^ per EMS agency. The institutional review board approved this study under expedited review.

### Study Setting and Population

Directors or STEMI coordinators from each EMS agency were asked to fill out a STEMI Regionalization Survey designed to evaluate the degree and duration of STEMI regionalization of care in each EMS jurisdiction. All 33 LEMSAs representing 58 counties in California responded and filled out the survey, resulting in a 100% response rate.

### STEMI Regionalization Survey

Rokos et al.^14^ provided a detailed approach on the development of the STEMI Regionalization Survey. The survey was developed to serve as an evidence-based assessment tool to evaluate and assess the degree and duration of STEMI regionalization of care for each EMS agency. The questions were categorized based on different elements of a STEMI regionalized system: availability of prehospital 12-lead ECG devices; destination protocols; designation of PCI-capable hospitals as STEMI Receiving Centers (SRC) that function 24/7; inter-hospital transfer protocols from non-PCI hospitals to SRCs; and hospital quality improvement (see Supplement for a copy of the survey). Each EMS agency was asked to identify the existence of each element as of 2014, as well as the year the element was implemented. As such, the survey represents the regionalization-of-care status for each EMS agency between 2006 (the first full year that clinical coding separated STEMI from non-STEMI) and 2014.

### Categories of Regionalization Status

For the years evaluated, the survey assessed the degree of each EMS agency’s regionalized status by providing four multiple-choice options aimed at capturing the level of regionalization reached for each of the elements above: 1) Level A (none- 0%); 2) Level B (some- <50%); 3) Level C (most- 50–94%); and Level D (all- ≥95%). We developed four categories to capture both the prehospital (EMS devices and destination protocols) and inter-hospital (non-PCI-capable referral hospitals) components of regionalization defined as follows: no regionalization; partial regionalization; substantial regionalization; and complete regionalization. Table 1 provides a summary of the regionalization category definitions.

## RESULTS

In 2006, only eight out of 58 counties (representing 42% of California’s STEMI population) had reached regionalization, with five counties (representing 13% of STEMI patients) considered completely regionalized (Table 1 and Figure). In 2011, the number of counties that had reached partial, substantial, and complete regionalization increased by 11, 14, and 13, respectively, from 2006. By 2014, all counties had at least one regionalization-of-care protocol in place; 30 out of 58 counties completely regionalized (representing 86% of STEMI patients), while the number of partially and substantially regionalized counties did not change from 2011 (Figure).

Of the counties that were not regionalized at baseline, 22 counties became partially regionalized, 12 became substantially regionalized, and 16 became completely regionalized in their first year of regionalization. The counties that became completely regionalized in their first year of regionalization generally were more populated than other counties. Not shown in the graph, a greater proportion of counties in Southern California reached complete regionalization by 2014, compared to Northern California, where the majority of counties reached only either partial or substantial regionalization. To collect the data we had to ensure the confidentiality of all counties and that no specific county would be identified in our results; thus, we are unable to provide maps of regionalization progress over time.

### Patient Demographic, Hospital, and County Trends

Table 2 shows a description of patient demographics by year of regionalization, where we considered “regionalized” as any level of regionalization (full descriptive statistics in Appendix Table 1). Subsequent sensitivity analyses using a more conservative definition of regionalization restricted to substantial or complete regionalization showed no changes. At baseline, already-regionalized communities compared to the whole sample had a slightly higher proportion of black (8% v. 5%), Hispanic (20% v. 16%), and Asian residents (10% v. 8%), and fewer White residents (58% v. 66%). While the proportion of Medicare-insured residents between earlier and later regionalized counties did not differ substantially, later regionalized counties had a higher proportion of privately insured residents (36% after 2011 vs. 33% before 2006), and earlier regionalized counties had a slightly higher proportion of Medicaid-insured patients (10% before 2006 v. 8% after 2011). Underlying co-morbidities of patients in earlier vs. later regionalized counties did not differ dramatically.

At the hospital level, earlier regionalized counties had a higher proportion of for-profit hospitals (22% before 2006 v. 11% after 2011), while later regionalized counties had a higher proportion of teaching hospitals (14% after 2011 v. 8% before 2006) and hospitals part of a system (88% after 2011 v. 72% before 2006). Additionally, counties that regionalized by 2006 had a Herfindahl-Hirschman Index (HHI, a measure of market concentration) mean of 0.14, indicating a low concentration of hospitals, 0.33 for counties regionalized by 2011, indicating a high concentration of hospitals, and 0.21 for counties that regionalized by 2014, indicating a moderate concentration of hospitals.

At the county level, communities that regionalized later had a slightly higher mean per capita income ($39,615) compared to those that regionalized earlier ($37,956), consistent with earlier regionalization trends in communities with higher proportions of Medicaid-insured individuals. However, we found no apparent trends in the percentage of the population identified as living under the poverty line, part of a racial/minority group, or elderly (> 65 years).

## DISCUSSION

STEMI regionalization and its impact have been particularly well-documented in the literature for North Carolina,^15^ and our study adds to that literature with the first description and documentation, to our knowledge, of regionalization-of-care efforts for STEMI patients in California. Our survey results cover years 2006–2014, showing that generally Southern California areas regionalized faster. In our description of patient and hospital characteristics in earlier vs. later regionalized counties, we found that while patient characteristics differed little, hospital characteristics varied to some degree. This is important in laying the groundwork for future analyses of the impact of regionalization on mortality outcomes.

### Factors of Early/Later Regionalization

#### Competition

Our descriptive characteristics of hospitals show that counties with low concentrations of hospitals regionalized earlier than regions with high or moderate concentrations of hospitals, suggesting that competition may be a deciding factor in how quickly a county can regionalize. Generally, it has been well documented that competition between hospitals can prevent a region from reaching full regionalization.^3^,^16^ Many hospitals within EMS agency catchment areas may be concerned with changes in patient volume, primarily due to potential misidentification of STEMI by prehospital personnel in patients with relevant symptomatology (e.g., chest pain, diaphoresis, shortness of breath), diverting potentially non-STEMI patients away from non-PCI-capable hospitals, which could result in losing additional revenue on top of revenue lost from STEMI patients being diverted away from non-PCI hospitals.^17^,^18^ Moreover, greater competition can exist in areas more concentrated with PCI-capable hospitals. Counties with a large number of available PCI-capable hospitals relative to the size of the county could have a lesser need for regionalization due to a higher likelihood of the nearby hospital having PCI capability.^19^

#### Administrative/structural differences in hospitals

Cardiac services such as PCI are considered one of the more profitable services a hospital can provide; therefore, it was not surprising when we found that generally, earlier regionalized counties had a greater percentage of for-profit hospitals. This could reflect the administrative and operational structural differences between for-profit and non-profit/government hospitals or the limitations facing non-profit/government hospitals to secure funding to become PCI-capable and regionalize. However, previous literature has suggested that our findings likely reveal the inclination of for-profit hospitals to offer PCI services and be motivated to regionalize faster as it would increase hospital revenue,^20^ suggesting that policymakers should look to increase incentives to prioritize increasing PCI services or regionalization of care, such as counting these activities as uncompensated care or requiring such capabilities as government regulations or for non-profit status.

#### Population Density

Population density could potentially act as a barrier to earlier regionalization. Counties with denser cities may have a lesser need as the closest hospital likely is PCI-capable, even if the number of available PCI-capable hospitals is relatively small. On the other hand, counties with more spread-out cities may have a greater need as more residents are likely to be located nearest to a small hospital, without PCI capability. We found that Southern California counties generally regionalized faster as they geographically tend to be more “spread out,” which provides some evidence that population density could be a potential factor in adopting regionalization protocols. Our findings suggest that regionalization could be more beneficial in lower population density areas, especially in areas with relatively larger rural populations. Although high population density areas may benefit less from regionalization, they could be more susceptible to reduced PCI access due to crowding, and may benefit more from increasing the number of PCI-capable hospitals in the area or PCI capability within the existing hospitals.^21^

#### Other Potential Factors

Other potential factors that we did not specifically study but were mentioned by EMS administrators and medical directors may have contributed to the pace and degree to which each county regionalized. For instance, while regionalization of care provided the primary impetus to improve compliance with the 2004 AHA guidelines requiring a door-to-balloon time within 90 minutes,^22^ purchasing prehospital ECG devices for all ambulances could be a substantial financial burden that EMS agencies cannot or do not want to bear as PCI-capable hospitals ultimately reap the financial rewards of adopting regionalization protocols.^21^,^23^ Factors not associated with EMS regulation, such as success obtaining external grants and private philanthropy for individual EMS agencies and availability of prehospital ECG devices, may have assisted with earlier regionalization of care,^24^ as reported by certain counties in Southern California. Furthermore, management factors such as “champions for change” and leadership influence in pushing for quality improvement may have also allowed for earlier regionalization, similar to quality improvement in other types of healthcare systems.^25^,^26^ On the other hand, conflicting and evolving literature on long-term outcome improvements offered by recognition of STEMI by field personnel and direct transport to PCI-capable SRCs could have delayed decisions to adopt regionalization protocols.^21^,^27^,^28^

Overall, examining trends in the regionalization of care has the potential to offer insight into how the reorganization of care can affect patient outcomes. These findings have important implications, as hospitals and EMS networks will require financial and organizational restructuring after adopting regionalization protocols. On a larger level, regionalized systems may allow providers and hospitals to benefit from reforms, such as the Medicare and CHIP Reauthorization Act of 2015,^29^ which has encouraged delivery systems to focus on value and quality, including access to timely cardiac care, rather than volume and public reporting.^30^

Our descriptive characteristics establish that earlier and later regionalized counties do not differ dramatically in patient characteristics, and their differences would therefore not be expected to confound our later analysis of mortality outcomes. Furthermore, while hospital characteristics do vary considerably, previous literature suggests that although hospital ownership may influence the willingness and rate of regionalization, patient outcomes do not differ widely between for-profit hospitals and non-profit hospitals.^31^ Future research studying the impact of regionalization of care for STEMI patients, as well as how and why certain systems had an easier time regionalizing early, may provide further insight into the impact and process of how such changes can be replicated in other settings.

## LIMITATIONS

Our study included several limitations. First, while we were able to receive responses to all questions from each EMS agency, we were only able to receive best approximate estimates in some responses of when each EMS agency reached a level of regionalization as in certain counties, the current administrator or medical director of the EMS agency might not have been present or have had knowledge of the state of cardiac care regionalization in 2005. Second, some directors and administrators could not provide records documenting the timeline of their protocols because these records did not exist or were erased. In these cases, we attempted to verify information by having external reviewers familiar with California’s regionalization trends examine the data and provide face validity checks for the provided responses. When questions arose for either limitation, we contacted the initial agency again and attempted to triangulate our information with other sources (e.g., previous administrators or medical directors, the AHA Mission Lifeline regional network directors and administrators) to obtain more accurate information. Last, our data only covered California, meaning that while our findings may provide general insight into the regionalization of care, they may not be entirely generalizable to other states across the nation.

## CONCLUSION

Our survey results allowed us to identify prehospital and inter-hospital elements within each EMS agency’s STEMI program to assess the degree, duration, and trends of their regionalization-of-care efforts. We identified hospital competition, hospital ownership, population density, and financial barriers to be some potential factors in slowing down or preventing complete regionalization of care. We did not find any dramatic differences in the underlying population characteristics based on regionalization-of-care status in our study period, providing some reassurance for future studies evaluating the impact of regionalization that any findings would be less likely due to differences in patient characteristics. Our findings allow providers and policymakers to recognize the barriers to establishing and potentially reorganizing regionalized systems of care, and may serve as a framework for continued regionalization of care in other regions.

## Supplementary Information





## Figures and Tables

**Figure:**
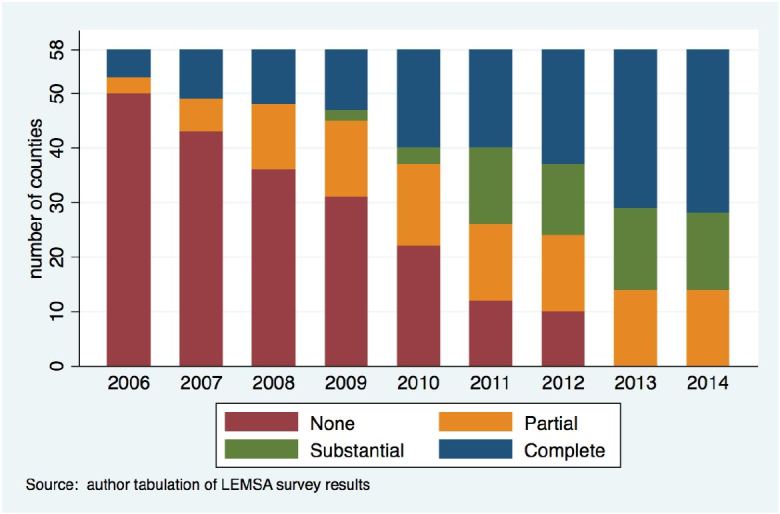
California STEMI regionalization status between 2006 and 2014. *STEMI*, ST-segment elevation myocardial infarction; *LEMSA*, local emergency medical service agency.

**Table 1:** ST-segment elevation myocardial infarction regionalization categories.

	None	Partial	Substantial	Complete
Criteria				
Prehospital protocols				
<50% coverage	X			
50–94% coverage		X (either)	X (both)	
≥95% coverage				X
Inter-hospital transfer protocols				
<50% coverage	X			
50–94% coverage		X (either)	X (both)	
≥95% coverage				X

None: Neither prehospital nor inter-hospital protocols were in place, or the protocols were implemented in less than 50% of the emergency medical service (EMS) agency jurisdiction.

Partial: Between 50% to 94% of the EMS agency jurisdiction had either prehospital or inter-hospital protocols in place, but not both.

Substantial: Between 50% to 94% of the EMS agency jurisdiction had both prehospital and inter-hospital protocols in place.

Complete: At least 95% of the EMS agency jurisdiction had both prehospital and inter-hospital protocols in place.

**Table 2:** Descriptive statistics at baseline (2006) by year of regionalization of care for STEMI patients in California.

	Whole sample		Regionalized as of 2006		Regionalized between 2007–11		Regionalized after 2011	
			
	N	%	N	%	N	%	N	%
Sex								
Female	6131	35%	2576	35%	2596	34%	959	35%
Male	11598	65%	4824	65%	4954	66%	1820	65%
Race/ethnicity								
White	11656	66%	4266	58%	5490	73%	1900	68%
Black	941	5%	556	8%	243	3%	142	5%
Hispanic	2784	16%	1463	20%	1050	14%	271	10%
Asian	1440	8%	735	10%	390	5%	315	11%
Other non-White races	908	5%	380	5%	377	5%	151	5%
Age distribution								
Less than 65	8140	46%	3349	45%	3425	45%	1366	49%
65 and above	9563	54%	4035	55%	4118	55%	1410	51%
65–69	1877	11%	765	10%	797	11%	315	11%
70–74	1729	10%	729	10%	759	10%	241	9%
75–79	1893	11%	803	11%	817	11%	273	10%
80–84	1877	11%	782	11%	828	11%	267	10%
85+	2187	12%	956	13%	917	12%	314	11%
Payment Categories								
Medicare	8909	50%	3610	49%	3952	52%	1347	48%
Medicaid	1380	8%	731	10%	440	6%	209	8%
Private Insurance	5870	33%	2424	33%	2452	32%	994	36%
Indigent	448	3%	159	2%	231	3%	58	2%
Self-pay	794	4%	360	5%	325	4%	109	4%
Other	328	2%	116	2%	150	2%	62	2%
Other admission hospital characteristics								
For profit	3078	17%	1636	22%	1124	15%	318	11%
Government	2180	13%	696	10%	1219	17%	265	10%
Teaching hospital	1457	9%	557	8%	520	7%	380	14%
Member of a system	13387	77%	5173	72%	5817	78%	2397	88%
Mean total beds in hospital (SD)	277	141	298	151	267	132	245	126
Mean occupancy rate (SD)	0.69	0.14	0.68	0.15	0.68	0.14	0.73	0.13
Mean HHI index (SD)	0.23	0.25	0.14	0.20	0.33	0.28	0.21	0.15
County characteristics								
Mean per capita income (SD)	$37,938	$10,516	$37,956	$8,950	$37,302	$9,606	$39,615	$15,418
% Population below poverty line (SD)	13	4.16	14	4.14	12	3.70	13	4.43
% Minority Population (SD)	22	8.71	25	6.90	18	7.86	27	10.21
% Population ≥ 65 years (SD)	11	1.77	10	0.76	11	2.09	11	2.22
Patient	17729		7400		7550		2779	
Population	36,457,548		16,260,460		14,755,954		5,441,135	
Counties	58		8		38		12	

*HHI,* Herfindahl-Hirschman Index*; SD*, standard deviation.
