# This Article Corrects: “Trends in Regionalization of Care for ST-Segment Elevation Myocardial Infarction”

**DOI:** 10.5811/westjem.2018.5.38435

**Published:** 2018-04-12

**Authors:** Renee Y. Hsia, Sarah Sabbagh, Nandita Sarkar, Karl Sporer, Ivan C. Rokos, John F. Brown, Ralph G. Brindis, Joanna Guo, Yu-Chu Shen

**Affiliations:** * University of California, San Francisco, Department of Emergency Medicine, San Francisco, California; † University of California, San Francisco, Philip R. Lee Institute for Health Policy Studies, San Francisco, California; ‡ National Bureau of Economic Research, Cambridge, Massachusetts; § Alameda County Emergency Medical Services Agency, Oakland, California; ¶ University of California, Los Angeles-Olive View Medical Center; Geffen School of Medicine, Los Angeles, California; || San Francisco Emergency Medical Services Agency, San Francisco, California; # University of California, San Francisco, Department of Medicine, San Francisco, California; ** Naval Postgraduate School, Graduate School of Business and Public Policy, Monterey, California

West J Emerg Med. 2017 October;18(6):1010–1017

Trends in Regionalization of Care for ST-Segment Elevation Myocardial Infarction.

Hsia RY, Sabbagh S, Sarkar N, Sporer K, Rokos IC, Brown JF, Brindis RG, Guo J, Shen Y

Erratum in

West J Emerg Med. 2018 May;19(3)630]. The authors would like to revise the *Conflict of Interest* statement on page 1016 to include grant award number R01HL114822.

PMCID: PMC5654868 [PubMed - indexed for MEDLINE]

