# Supplementary material for: Trends in Regionalization of Care for ST-Segment Elevation Myocardial Infarction
Source: West J Emerg Med. 2017 Sep 11;18(6):1010–7. doi: 10.5811/westjem.2017.8.34592 (PMC5654868; doi:10.5811/westjem.2017.8.34592)
Supplement: Supplementary file 1 [file wjem-18-1010-s001.docx]

**Appendix Table 1. Full descriptive statistics at baseline (2006) by year of regionalization**

|  | | **Whole**  **Sample** | | | **Regionalized as of 2006** | | | | **Regionalized between 2007-2011** | | | | | | | **Regionalized after 2011** | | |
| --- | --- | --- | --- | --- | --- | --- | --- | --- | --- | --- | --- | --- | --- | --- | --- | --- | --- | --- |
|  | **N** | | **%** | **N** | | | **%** | | **N** | | **%** | | **N** | | | | | **%** |
| **Patient demographics** |  | |  |  | | |  | |  |  | |  | | | | |  |  |
| Female | 6131 | | 35% | 2576 | | | 35% | | 2596 | | 34% | | 959 | | | | | 35% |
| White | 11656 | | 66% | 4266 | | | 58% | | 5490 | | 73% | | 1900 | | | | | 68% |
| Black | 941 | | 5% | 556 | | | 8% | | 243 | | 3% | | 142 | | | | | 5% |
| Hispanic | 2784 | | 16% | 1463 | | | 20% | | 1050 | | 14% | | 271 | | | | | 10% |
| Asian | 1440 | | 8% | 735 | | | 10% | | 390 | | 5% | | 315 | | | | | 11% |
| Other non-white races | 908 | | 5% | 380 | | | 5% | | 377 | | 5% | | 151 | | | | | 5% |
| Age distribution |  | |  |  | | |  | |  | |  | |  | | | | |  |
| Less than 65 | 8140 | | 46% | 3349 | | | 45% | | 3425 | | 45% | | 1366 | | | | | 49% |
| 65 and above | 9563 | | 54% | 4035 | | | 55% | | 4118 | | 55% | | 1410 | | | | | 51% |
| 65–69 | 1877 | | 11% | 765 | | | 10% | | 797 | | 11% | | 315 | | | | | 11% |
| 70–74 | 1729 | | 10% | 729 | | | 10% | | 759 | | 10% | | 241 | | | | | 9% |
| 75–79 | 1893 | | 11% | 803 | | | 11% | | 817 | | 11% | | 273 | | | | | 10% |
| 80–84 | 1877 | | 11% | 782 | | | 11% | | 828 | | 11% | | 267 | | | | | 10% |
| 85+ | 2187 | | 12% | 956 | | | 13% | | 917 | | 12% | | 314 | | | | | 11% |
| **Payment Categories** |  | |  |  | | |  | |  | |  | |  | | | |  | |
| Medicare | 8909 | | 50% | 3610 | | | 49% | | 3952 | | 52% | | 1347 | | | | | 48% |
| Medicaid | 1380 | | 8% | 731 | | | 10% | | 440 | | 6% | | 209 | | | | | 8% |
| Private Insurance | 5870 | | 33% | 2424 | | | 33% | | 2452 | | 32% | | 994 | | | | | 36% |
| Indigent | 448 | | 3% | 159 | | | 2% | | 231 | | 3% | | 58 | | | | | 2% |
| Self-pay | 794 | | 4% | 360 | | | 5% | | 325 | | 4% | | 109 | | | | | 4% |
| Other | 328 | | 2% | 116 | | | 2% | | 150 | | 2% | | 62 | | | | | 2% |
| **Patient comorbid conditions** |  | |  |  | | |  | |  | |  | |  | | | | |  |
| Peripheral vascular disease | 1434 | | 8% | 588 | | | 8% | | 618 | | 8% | | 228 | | | | | 8% |
| Pulmonary Circulation disorders | 422 | | 2% | 151 | | | 2% | | 191 | | 3% | | 80 | | | | | 3% |
| Diabetes (uncomplicated + complicated) | 5413 | | 31% | 2414 | | | 33% | | 2193 | | 29% | | 806 | | | | | 29% |
| Renal failure | 2036 | | 11% | 905 | | | 12% | | 799 | | 11% | | 332 | | | | | 12% |
| Liver disease | 223 | | 1% | 95 | | | 1% | | 91 | | 1% | | 37 | | | | | 1% |
| Cancer | 469 | | 3% | 195 | | | 3% | | 204 | | 3% | | 70 | | | | | 3% |
| Dementia | 445 | | 3% | 209 | | | 3% | | 183 | | 2% | | 53 | | | | | 2% |
| Valvular disease | 1577 | | 9% | 616 | | | 8% | | 699 | | 9% | | 262 | | | | | 9% |
| Hypertension (uncomplicated + complicated) | 11220 | | 63% | 4818 | | | 65% | | 4673 | | 62% | | 1729 | | | | | 62% |
| Chronic pulmonary disease | 2786 | | 16% | 1123 | | | 15% | | 1231 | | 16% | | 432 | | | | | 16% |
| Rheumatoid arthritis/collagen vascular | 316 | | 2% | 119 | | | 2% | | 154 | | 2% | | 43 | | | | | 2% |
| Coagulation deficiency | 527 | | 3% | 232 | | | 3% | | 211 | | 3% | | 84 | | | | | 3% |
| Obesity | 1775 | | 10% | 704 | | | 10% | | 765 | | 10% | | 306 | | | | | 11% |
| Substance abuse | 837 | | 5% | 305 | | | 4% | | 384 | | 5% | | 148 | | | | | 5% |
| Depression | 808 | | 5% | 314 | | | 4% | | 351 | | 5% | | 143 | | | | | 5% |
| Psychosis | 305 | | 2% | 152 | | | 2% | | 114 | | 2% | | 39 | | | | | 1% |
| Hypothyroidism | 1403 | | 8% | 545 | | | 7% | | 659 | | 9% | | 199 | | | | | 7% |
| Paralysis and other neurological disorder | 1211 | | 7% | 539 | | | 7% | | 491 | | 7% | | 181 | | | | | 7% |
| Chronic peptic ulcer disease | 10 | | 0% | 5 | | | 0% | | 1 | | 0% | | 4 | | | | | 0% |
| Weight loss | 219 | | 1% | 103 | | | 1% | | 88 | | 1% | | 28 | | | | | 1% |
| Fluid and electrolyte disorders | 2728 | | 15% | 1186 | | | 16% | | 1126 | | 15% | | 416 | | | | | 15% |
| Anemia (blood loss and deficiency) | 2562 | | 14% | 1146 | | | 15% | | 1006 | | 13% | | 410 | | | | | 15% |
| **Other admission hospital characteristics** |  | |  |  | | |  | |  | |  | |  | | | | |  |
| For profit | 3078 | | 17% | 1636 | | | 22% | | 1124 | | 15% | | 318 | | | | | 11% |
| Government | 2180 | | 13% | 696 | | | 10% | | 1219 | | 17% | | 265 | | | | | 10% |
| Teaching hospital | 1457 | | 9% | 557 | | | 8% | | 520 | | 7% | | 380 | | | | | 14% |
| Member of a system | 13387 | | 77% | 5173 | | | 72% | | 5817 | | 78% | | 2397 | | | | | 88% |
| Mean total beds in hospital (SD) | 277 | | 141 | 298 | | | 151 | | 267 | | 132 | | 245 | | | | | 126 |
| Mean occupancy rate (SD) | 0.69 | | 0.14 | 0.68 | | | 0.15 | | 0.68 | | 0.14 | | 0.73 | | | | | 0.13 |
| Mean HHI index (SD) | 0.23 | | 0.25 | 0.14 | | | 0.20 | | 0.33 | | 0.28 | | 0.21 | | | | | 0.15 |
| **County characteristics** |  | |  |  | | |  | |  |  | |  | |  | | | |  |
| Mean per capita income (SD) | $37,938 | | $10,516 | $37,956 | | | $8,950 | | $37,302 | | $9,606 | | | | | $39,615 | | $15,418 |
| % Population below poverty line (SD) | 13 | | 4.16 | 14 | | | 4.14 | | 12 | | 3.70 | | | | | 13 | | 4.43 |
| % Minority Population (SD) | 22 | | 8.71 | 25 | | | 6.90 | | 18 | | 7.86 | | | | | 27 | | 10.21 |
| % Population ≥ 65 years (SD) | 11 | | 1.77 | 10 | | | 0.76 | | 11 | | 2.09 | | | | | 11 | | 2.22 |
| Patient | 17729 | | |  | | 7400 | |  | 7550 | | | | | | 2779 | | | |
| Population | 36,457,548 | | | 16,260,460 | | | | | 14,755,954 | | | | | | 5,441,135 | | | |
| Counties | 58 | | |  | | 8 | |  | 38 | | | | | | 12 | | | |

**STEMI Regionalization Survey**

**Purpose:** This survey is part of a project funded by the National Institutes of Health/National Heart, Lung, and Blood Institute (1R56HL121108-01A1) entitled, “The Impact of Cardiac Care Regionalization on Access, Treatment, and Outcomes,” which will evaluate the impact of STEMI regionalization on patient outcomes.

**Background**:

- This 8-item survey quantitatively evaluates the Degree and Duration of STEMI regionalization in your EMS jurisdiction.
- This survey does *not* evaluate the Quality or Efficiency of STEMI care in a region.

***Instructions:***

- *Please circle ONE choice that most accurately reflects your Region’s status in 2014.*
- *If you select choice C, please enter calendar year after the arrow.*
- *Whenever you select choice D, enter calendar year milestones for both C and D.*
- *When unable to answer a question with perfect accuracy, your best approximation is acceptable.*

1. **Pre-hospital 12-lead Electrocardiogram (PH-ECG):** In 2014, approximately what proportion of EMS provider teams (either primary or secondary responders) are equipped with a 12-lead ECG device and routinely acquire a pre-hospital ECG when assessing 9-1-1 patients with symptoms suggestive of acute cardiac ischemia?
   1. None (0%)
   2. Some (< 50%)
   3. Most (50% - 94%) **🡪** In what year did you reach this level? ________ Exact month (if known)? _____
   4. All (≥ 95%) **🡪** In what year did you reach this level? ________ Exact month (if known)? _____
   5. Do not know or track this information
2. **Destination Protocols**: In 2014, approximately what proportion of EMS providers are authorized by protocol to bypass nearby non-PCI-hospitals when transporting PH-ECG identified STEMI patients and instead drive further (if needed) to the most appropriate STEMI Receiving Center (PCI-capable hospital)?
   1. None (0%)
   2. Some (< 50%)
   3. Most (50% - 94%) **🡪** In what year did you reach this level? ________ Exact month (if known)? _____
   4. All (≥ 95%) **🡪** In what year did you reach this level? ________ Exact month (if known)? _____
   5. Do not know or track this information
3. **PCI-capable hospitals**: In 2014, approximately what proportion of PCI-capable hospitals in your region are designated STEMI Receiving Centers (SRC) by authority of the Local EMS Agency (LEMSA)?

*(Note: an SRC is available 24/7 and accepts STEMI patients even when the hospital is on ambulance diversion due to ED saturation).*

- 1. None (0%)
  2. Some (< 50%)
  3. Most (50% - 94%) **🡪** In what year did you reach this level? ________ Exact month (if known)? _____
  4. All (≥ 95%) **🡪** In what year did you reach this level? ________ Exact month (if known)? _____
  5. Do not know or track this information

1. **Daytime PCI-hospitals:** In 2014, does your region contain any hospitals that only have PCI-capability weekdays during regular daytime working hours?

____ Yes ____ No

**If Yes** in 2014, approximately what proportion of these daytime PCI-hospitals emergently transfer walk-in STEMI patients arriving after-hours (nights and weekends) to a designated SRC?

- 1. None (0%)
  2. Some (< 50%)
  3. Most (50% - 94%) **🡪** In what year did you reach this level? ________ Exact month (if known)? _____
  4. All (≥ 95%) **🡪** In what year did you reach this level? ________ Exact month (if known)? _____
  5. Do not know or track this information

1. **Non-PCI-capable Type 1 Referral Hospitals:** In 2014, approximately what proportion of non-PCI-capable hospitals routinely transfers patients to a designated SRC for emergent primary PCI?

*(Note: For this survey, potential Type 1 Referral Hospitals are either ≤30 miles or ≤30 minute drive (or fly) time from the SRC so that the 120-minute Guideline benchmark is realistically achievable).*

- 1. None (0%)
  2. Some (< 50%)
  3. Most (50% - 94%) **🡪** In what year did you reach this level? ________ Exact month (if known)? _____
  4. All (≥ 95%) **🡪** In what year did you reach this level? ________ Exact month (if known)? _____
  5. Do not know or track this information

1. **Non-PCI-capable Type 2 Referral Hospitals:** In 2014, approximately what proportion of non-PCI-capable hospitals first treats STEMI patients with pre-transfer Fibrinolytics (unless contraindicated) and then immediately transfers patients to a designated SRC for either Rescue PCI or Non-emergent PCI as clinically indicated?

*(Note: For this survey, potential Type 2 Referral Hospitals are either >30 miles or >30 minutes drive (or fly) time from the SRC so that the 120-minute Guideline benchmark is generally unrealistic).*

- 1. None (0%)
  2. Some (< 50%)
  3. Most (50% - 94%) **🡪** In what year did you reach this level? ________ Exact month (if known)? _____
  4. All (≥ 95%) **🡪** In what year did you reach this level? ________ Exact month (if known)? _____
  5. Do not know or track this information

1. **Quality Improvement for STEMI Receiving Centers:** In 2014, approximately what proportion of PCI-capable Hospitals submits time-to-treatment data as part of a QI program providing Regional Results to the LEMSA?
   1. None (0%)
   2. Some (< 50%)
   3. Most (50% - 94%) **🡪** In what year did you reach this level? ________ Exact month (if known)? _____
   4. All (≥ 95%) **🡪** In what year did you reach this level? ________ Exact month (if known)? _____
   5. Do not know or track this information
2. **Quality Improvement for Referral Hospitals (both Type 1 & 2):** In 2014, approximately what proportion of non-PCI-capable Hospitals submits time-to-transfer data as part of a QI program providing Regional Results to the LEMSA?

*(Note: These Referral Hospitals can either directly submit data themselves or indirectly rely upon their partnering STEMI Receiving Center to submit data on their behalf).*

- 1. None (0%)
  2. Some (< 50%)
  3. Most (50% - 94%) **🡪** In what year did you reach this level? ________ Exact month (if known)? _____
  4. All (≥ 95%) **🡪** In what year did you reach this level? ________ Exact month (if known)? _____
  5. Do not know or track this information

**Please free-text comments or survey suggestions below:**

*Please send your responses either by: (1) email to:* [*sarah.sabbagh@ucsf.edu*](mailto:sarah.sabbagh@ucsf.edu)*; (2) regular mail to: Sarah Sabbagh, c/o Dr. Renee Hsia, San Francisco General Emergency Department, 1001 Potrero Ave, 1E21, San Francisco, CA 94110; or (3) fax: Attn: Sarah Sabbagh, (415) 206-5818. If you have any questions, please contact Sarah via email (see above) or phone at (415) 206-4612.*
