# Supplementary material for: Trends in Regionalization of Care for ST-Segment Elevation Myocardial Infarction
Source: West J Emerg Med. 2017 Sep 11;18(6):1010–7. doi: 10.5811/westjem.2017.8.34592 (PMC5654868; doi:10.5811/westjem.2017.8.34592)
Supplement: Supplementary file 2 [file wjem-18-1010-s002.docx]

**Purpose:** This survey is part of a project funded by the National Institutes of Health/National Heart, Lung, and Blood Institute (1R56HL121108-01A1) entitled, “The Impact of Cardiac Care Regionalization on Access, Treatment, and Outcomes,” which will evaluate the impact of STEMI regionalization on patient outcomes.

**Background**:

- This 8-item survey quantitatively evaluates the Degree and Duration of STEMI regionalization in your EMS jurisdiction.
- This survey does *not* evaluate the Quality or Efficiency of STEMI care in a region.

***Instructions:***

- *Please circle ONE choice that most accurately reflects your Region’s status in 2014.*
- *If you select choice C, please enter calendar year after the arrow.*
- *Whenever you select choice D, enter calendar year milestones for both C and D.*
- *When unable to answer a question with perfect accuracy, your best approximation is acceptable.*

1. **Pre-hospital 12-lead Electrocardiogram (PH-ECG):** In 2014, approximately what proportion of EMS provider teams (either primary or secondary responders) are equipped with a 12-lead ECG device and routinely acquire a pre-hospital ECG when assessing 9-1-1 patients with symptoms suggestive of acute cardiac ischemia?
   1. None (0%)
   2. Some (< 50%)
   3. Most (50% - 94%) **🡪** In what year did you reach this level? ________ Exact month (if known)? _____
   4. All (≥ 95%) **🡪** In what year did you reach this level? ________ Exact month (if known)? _____
   5. Do not know or track this information
2. **Destination Protocols**: In 2014, approximately what proportion of EMS providers are authorized by protocol to bypass nearby non-PCI-hospitals when transporting PH-ECG identified STEMI patients and instead drive further (if needed) to the most appropriate STEMI Receiving Center (PCI-capable hospital)?
   1. None (0%)
   2. Some (< 50%)
   3. Most (50% - 94%) **🡪** In what year did you reach this level? ________ Exact month (if known)? _____
   4. All (≥ 95%) **🡪** In what year did you reach this level? ________ Exact month (if known)? _____
   5. Do not know or track this information
3. **PCI-capable hospitals**: In 2014, approximately what proportion of PCI-capable hospitals in your region are designated STEMI Receiving Centers (SRC) by authority of the Local EMS Agency (LEMSA)?

*(Note: an SRC is available 24/7 and accepts STEMI patients even when the hospital is on ambulance diversion due to ED saturation).*

- 1. None (0%)
  2. Some (< 50%)
  3. Most (50% - 94%) **🡪** In what year did you reach this level? ________ Exact month (if known)? _____
  4. All (≥ 95%) **🡪** In what year did you reach this level? ________ Exact month (if known)? _____
  5. Do not know or track this information

1. **Daytime PCI-hospitals:** In 2014, does your region contain any hospitals that only have PCI-capability weekdays during regular daytime working hours?

____ Yes ____ No

**If Yes** in 2014, approximately what proportion of these daytime PCI-hospitals emergently transfer walk-in STEMI patients arriving after-hours (nights and weekends) to a designated SRC?

- 1. None (0%)
  2. Some (< 50%)
  3. Most (50% - 94%) **🡪** In what year did you reach this level? ________ Exact month (if known)? _____
  4. All (≥ 95%) **🡪** In what year did you reach this level? ________ Exact month (if known)? _____
  5. Do not know or track this information

1. **Non-PCI-capable Type 1 Referral Hospitals:** In 2014, approximately what proportion of non-PCI-capable hospitals routinely transfers patients to a designated SRC for emergent primary PCI?

*(Note: For this survey, potential Type 1 Referral Hospitals are either ≤30 miles or ≤30 minute drive (or fly) time from the SRC so that the 120-minute Guideline benchmark is realistically achievable).*

- 1. None (0%)
  2. Some (< 50%)
  3. Most (50% - 94%) **🡪** In what year did you reach this level? ________ Exact month (if known)? _____
  4. All (≥ 95%) **🡪** In what year did you reach this level? ________ Exact month (if known)? _____
  5. Do not know or track this information

1. **Non-PCI-capable Type 2 Referral Hospitals:** In 2014, approximately what proportion of non-PCI-capable hospitals first treats STEMI patients with pre-transfer Fibrinolytics (unless contraindicated) and then immediately transfers patients to a designated SRC for either Rescue PCI or Non-emergent PCI as clinically indicated? *(Note: For this survey, potential Type 2 Referral Hospitals are either >30 miles or >30 minutes drive (or fly) time from the SRC so that the 120-minute Guideline benchmark is generally unrealistic).*
   1. None (0%)
   2. Some (< 50%)
   3. Most (50% - 94%) **🡪** In what year did you reach this level? ________ Exact month (if known)? _____
   4. All (≥ 95%) **🡪** In what year did you reach this level? ________ Exact month (if known)? _____
   5. Do not know or track this information
2. **Quality Improvement for STEMI Receiving Centers:** In 2014, approximately what proportion of PCI-capable Hospitals submits time-to-treatment data as part of a QI program providing Regional Results to the LEMSA?
   1. None (0%)
   2. Some (< 50%)
   3. Most (50% - 94%) **🡪** In what year did you reach this level? ________ Exact month (if known)? _____
   4. All (≥ 95%) **🡪** In what year did you reach this level? ________ Exact month (if known)? _____
   5. Do not know or track this information
3. **Quality Improvement for Referral Hospitals (both Type 1 & 2):** In 2014, approximately what proportion of non-PCI-capable Hospitals submits time-to-transfer data as part of a QI program providing Regional Results to the LEMSA?

*(Note: These Referral Hospitals can either directly submit data themselves or indirectly rely upon their partnering STEMI Receiving Center to submit data on their behalf).*

- 1. None (0%)
  2. Some (< 50%)
  3. Most (50% - 94%) **🡪** In what year did you reach this level? ________ Exact month (if known)? _____
  4. All (≥ 95%) **🡪** In what year did you reach this level? ________ Exact month (if known)? _____
  5. Do not know or track this information

**Please free-text comments or survey suggestions below:**

*Please send your responses either by: (1) email to:* [*sarah.sabbagh@ucsf.edu*](mailto:sarah.sabbagh@ucsf.edu)*; (2) regular mail to: Sarah Sabbagh, c/o Dr. Renee Hsia, San Francisco General Emergency Department, 1001 Potrero Ave, 1E21, San Francisco, CA 94110; or (3) fax: Attn: Sarah Sabbagh, (415) 206-5818. If you have any questions, please contact Sarah via email (see above) or phone at (415) 206-4612.*
